# Pleomorphic giant cell carcinoma of the prostate: clinicopathologic analysis and oncological outcomes

**DOI:** 10.1007/s00428-022-03481-7

**Published:** 2023-01-05

**Authors:** Andreia Bilé-Silva, Antonio Lopez-Beltran, Henrique Rasteiro, Nuno Vau, Ana Blanca, Enrique Gomez, Frederico Gaspar, Liang Cheng

**Affiliations:** 1 Urology Department, Egas Moniz Hospital, Centro Hospitalar de Lisboa Ocidental, Lisbon, Portugal; 2 Pathology Department, Champalimaud Clinical Centre, Lisbon, Portugal; 3 Department of Morphological Sciences, Cordoba University Medical School, Cordoba, Spain; 4 Medical Oncology, Champalimaud Clinical Centre, Lisbon, Portugal; 5 Department of Urology, Maimonides Institute of Biomedical Research of Cordoba (IMIBIC), Reina Sofia University Hospital (HURS), Cordoba, 14004 Spain; 6 Department of Pathology and Laboratory Medicine, Brown University Medical School, Lifespan Academic Medical Center, Providence, RI 02903 USA

**Keywords:** Prostate cancer, Pleomorphic giant cell carcinoma, Grade group 5, ISUP 5, Hormone-resistance, Castration-resistant

## Abstract

We report on the clinicopathologic features of 27 pleomorphic giant cell carcinoma (PGCC) cases of the prostate identified in 20 patients with an age range of 51 to 84 years (68 ± 9; median 71 years). Charlson comorbidity index ranged from 3 to 12. Serum PSA ranged from 4.30 to 662 ng/mL (median 13 ng/mL). On histologic examination, bizarre giant cells with pleomorphic nuclei characterized pleomorphic giant cell carcinoma of the prostate. PGCC component was present in 5% to 100%, with half of the patients presenting with ≥ 20%. Half of the patients initially presented with T4 and 26% with T3 disease. All patients were considered Gleason scores of 9 to 10 (ISUP grade 5). A combination of hormone therapy with chemotherapy with or without radiation therapy was applied in 68% of patients. On follow-up, 14 patients (52%) were alive with disease (1–69 months) or dead of disease (1–38 months). Patients diagnosed earlier with lower TNM stage had longer survival than those diagnosed at a later T-stage or with metastatic disease (*p* = 0.02). The percentage of PGCC was not related to survival in the current study. Molecular alterations in 3 samples showed a microsatellite-stable disease with low tumor mutation burden and variable PTEN, PTCH1, KDM6A, ARv7, and PIK3CA loss/alteration, TP53 mutation, TMPRSS2-ERG fusion, and MYC, PIK3CB, RICTOR, or IRS2 amplification. Our findings suggest that PGCC is a rare and aggressive subtype of prostate carcinoma whose recognition may steer clinicians to adopt more aggressive treatments and investigate new therapeutic strategies.

## Introduction 

Pleomorphic giant cell carcinoma (PGCC) is a rare histologic subtype of acinar adenocarcinoma of the prostate (PCa) recognized by the World Health Organization (WHO) classification of genitourinary tumors [1]. Available data support PGCC as an aggressive form of PCa that displays a dismal prognosis, despite therapy [1–10]. Early studies place PGCC within the spectrum of Gleason pattern 5 [6, 11–17]. A PubMed database literature search (www.pubmed.gov) identified 51, well-illustrated previously reported examples, most of them with a limited focal representation of the pleomorphic giant cell component [1–24]. Alharbi et al. set 5% as a minimum requirement to qualify for PGCC and reported this uncommon subtype as prostate adenocarcinoma with focal pleomorphic giant cell features [2].

Giant bizarre cells with pleomorphic nuclei typically characterize PGCC on conventional histologic examination [1, 5, 6, 9, 14]. PGCC may be associated with acinar adenocarcinoma of the prostate and with other rare histologic subtypes of prostate carcinomas such as ductal adenocarcinoma and squamous, sarcomatoid, or neuroendocrine carcinomas [1]. When associated with conventional prostate carcinoma, the latter typically shows features of high Gleason grade that falls currently into the ISUP grade group 5 [2, 5, 6, 9], as recently recognized by the 5th edition of the WHO classification of urinary and male genital tumors [1].

Pleomorphic giant cell carcinoma is frequently seen in the clinical context of patients that have received previous PCa-directed treatments, typically androgen deprivation therapy [3–5, 8, 10, 12, 24]. Despite the limited data available, PGCC is more frequently observed in the context of metastatic disease. The fact that makes PGCC a diagnostic challenge is due to the known existence of pleomorphic giant cell carcinomas in the lungs, urinary bladder, and pancreas, among other organs [1, 6, 13, 15, 17, 21, 23]. Therefore, the diagnostic of PGCC is frequently preceded by variable immunohistochemical investigation to assess the primary origin [2]. Typically, prostate lineage markers such as PSA, NKX3.1, PSMA, or androgen receptors are often applied to solve this challenge. The clinical context, with a history of PCa, results extremely helpful during the workup of these patients.

Limited available molecular data based on less than 20 interrogated cases identified a variety of PTEN and TP53 alterations, BRCA2, PIK3CA, and BRAF mutations and rare TMPRSS2-ERG fusion or SND1-BRAF fusion as the genomic signature of PGCC [5, 7, 11].

The current study aims to report on the clinicopathologic features and oncological outcomes of a prospectively identified cohort series of 27 cases of PGCC diagnosed in 20 patients treated at our institution. Available molecular features in three patients are also reported together with a thorough literature review. Even if rare, prompt recognition of PGCC may steer clinicians to adopt more aggressive treatments and investigate new therapeutic strategies.

## Materials and methods

An observational study based on a prospectively maintained database was conducted. A total of 27 cases from 20 patients diagnosed with PGCC of the prostate were retrieved from the archive of Pathology of Champalimaud Cancer Centre, a comprehensive cancer center in Lisbon, Portugal. Available clinical information was obtained from the patient’s medical record, and an average of seventeen (range 3–40) H&E-stained slides from routinely formalin-fixed, paraffin-embedded material from each case was systematically re-evaluated by one specialized uropathologist (ALB), who identified PGCC cases to be included in the database.

The histologic evaluation included the assessment of the Gleason score at initial diagnosis and assessment of PGCC presence and percentage in each diagnostic sample. Giant bizarre cells with pleomorphic nuclei identified on H&E-stained glass slides typically characterized PGCC. A Gleason pattern 5 was assigned to PGCC upon diagnosis. Additional clinicopathologic features recorded during evaluation included perineural and/or lymphovascular invasion, cribriform architecture, and/or intraductal carcinoma or tumor necrosis. The histologic classification of the tumors followed the 2022 revision of the World Health Organization Classification of Urinary and Male Genital organs [1]. The percentage of pleomorphic giant cell carcinoma in each case was recorded. For analysis, the case series was then split according to the percentage of pleomorphic giant cell carcinoma. The cases spanned a period of 7 years with the earliest case diagnosed in 2014 and the last one in 2021, thus resulting in a follow-up of 1–69 months (mean ± standard deviation 24 ± 23 months; median 18 months). Patients’ demographics and comorbidities were also assessed, as well as the PSA level and AJCC/TNM (8th edition) [25] category at primary diagnosis, the treatment(s) received prior to or following the diagnosis of PGCC, and the clinical outcome.

Immunohistochemistry was performed on selected representative 4-µm-thick paraffin sections to solve specific differential diagnostic considerations and included PSA (clone 35H9, prediluted), PSMA (clone A16-4, 1/50 dilution), AR (clone SP107, prediluted), NKX3.1 (clone EP356, prediluted), ERG (clone EPR3864, prediluted), Ki-67 (clone K2, prediluted), p53 (clone DO-7, prediluted), synaptophysin (Leica, clone 27G12, prediluted), p63 (clone 7JUL, prediluted), GATA-3 (Cell Marque, clone L50–823, prediluted), PDL1 (assay 22C3), CK AE1/AE3 (clone AE1/AE3, prediluted), racemase (clone EPMU1, prediluted), CK20 (clone Ks20.8, prediluted), CK7 (clone RN7, prediluted), CK5/6 (clone D5/16B4, prediluted), CD138 (clone MI15, prediluted), INI1 (clone MRQ-27, prediluted), e-cadherin (clone 36B5, prediluted), p120 (clone EP66, prediluted), -hCG (Leica, polyclonal, prediluted), inhibin (clone R1, prediluted), and p40 (clone BC28, prediluted). Immunohistochemistry followed standard protocols for a given antibody, using either Ventana BenchMark or Leica Bond platforms. Appropriated negative and positive controls were included in every run.

Molecular analysis based on Foundation One CDx® (Roche Diagnostics, Penzberg, Germany) was performed on patients 13 (case 17) and 15 (case 20); OncoDEEP® (OncoDNA, Brussels, Belgium) was done in an additional metastatic biopsy sample of patient 13 (case 18).

### Statistical analysis

Categorical variables were presented as frequencies or percentages. Continuous variables were reported as mean ± standard deviation. The Kaplan–Meier method was used to estimate the distribution of survival separately for the patients diagnosed with different percentages of pleomorphic giant cell component or disease stage and to compare differences related to therapy prior to or after PGCC diagnosis. Statistical analysis was performed using IBM‐SPSS v.25 for Windows (IBM Corp, Armonk, NY). Statistically significant results were considered as *p* inferior to 0.05.

## Results

Table 1 summarizes the main clinicopathological features of the 27 cases of PGCC of cancer diagnosed in 20 patients. Patients ranged in age from 51 to 84 years (68 ± 9; median 71 years). Charlson comorbidity index of 8 ± 3 was obtained. PSA at initial diagnosis ranged from 4.3 to 662 ng/mL (median 13 ng/mL). The patient stage was IIIA or above, with 63% (12/20) of the patients in a stage IVB ab initio.

**Table 1:** Demographic characteristics and clinicopathologic features of pleomorphic giant cell carcinoma (PGCC) series

Patient #	Case #	Age (years)	Charlson comorbidity index	iPSA (ng/mL)	Date of initial diagnosis	Gleason score at initial diagnosis^*^	AJCC/TNM at initial diagnosis^†^	Treatment(s) prior to PGCC diagnosis	Date of PGCC diagnosis	Time from initial diagnosis to PGCC^‡^ (mo)	PGCC %, Gleason score and sample	PGCC pathologic features	PGCC treatment(s) after diagnosis	Clinical outcome (mo)^§^
1	1	72	10	13.00	01/11/2012	5 + 4	M1b (IVB)	HT	28/07/2014	20	5%; 5 + 4; PBx	LVI, PNI, cribriform	HT	DOD (1)
2	2	75	5	13.70	26/01/2015	5 + 4	M0 (-)	-	26/01/2015	0	5%; 5 + 4; PBx^**^	PNI	HT + RT, HT	AWD (66)
3	3	62	8	662.00	26/06/2014	4 + 5	cT2bN1M1c (IVB)	HT + CT	04/06/2015	11	20%; 5 + 5; TURP	LVI, PNI, 20% necrosis	HT + CT	DOD (2)
	4							HT + CT	22/07/2015	12	20%; 5 + 5; TURBT	LVI, 25% necrosis	HT + CT	
4	5	84	12	6.00	06/08/2015	5 + 5	cT4N1M1b (IVB)	-	06/08/2015	0	10%; 5 + 5; PBx^**^	PNI, 5% necrosis, MiNEN	HT + CT + ICI	DOD (24)
5	6	51	8	43.00	07/08/2014	4 + 4	M1c (IVB)	HT	21/10/2015	14	60%; 5 + 5; TURP	PNI	HT + CT	AWD (30)
	7							HT + CT	06/06/2017	33	60%; 5 + 5; PBx	PNI	HT + CT + ICI	
6	8	70	10	6.00	01/05/2005	3 + 4	M1c (IVB)	HT	02/03/2016	130	10%; 5 + 5; TURP	PNI	CT + Ra-233	DOD (38)
7	9	73	9	67.00	01/01/2013	3 + 4	M1b (IVB)	HT + CT	03/03/2016	38	10%; 5 + 5; TURP	PNI	HT + CT	AWD (14)
	10							HT + CT	01/03/2017	50	40%; 5 + 5; TURP	LVI, PNI,5% necrosis	HT + CT	
8	11	74	10	22.60	01/07/2014	4 + 5	cT4N1M1c (IVB)	HT	08/04/2016	21	10%; 5 + 4; TURP	PNI	HT + CT	AWD (40)
9	12	56	4	5.95	15/04/2016	4 + 4	pT3aN0M0 (IIIB)	-	15/04/2016	0	5%; 5 + 4; PBx^**^	PNI, IDCP	RPLND	AWD (69)
	13							-	23/06/2016	2	5%; 4 + 5; RPLND	PNI	sRT	
10	14	70	6	6.50	11/10/2016	4 + 4	pT3bN0M0 (IIIB)	-	11/01/2017	3	20%; 5 + 5; RPLND	IDCP	HT	AWD (47)
11	15	54	3	5.22	01/07/2016	4 + 4	pT3bN0M0 (IIIB)	-	08/11/2016	4	5%; 5 + 5; RPLND	LVI, PNI, IDCP	RPLND, HT	AWD (63)
12	16	72	5	N/A	14/02/2017	4 + 4	cT2bN0M0 (IIIA)	-	14/02/2017	0	5%; 5 + 4; PBx^**^	LVI, IDCP	HT + RT, HT	AWD (45)
13	17	64	9	75.34	13/11/2017	5 + 5	cT4N1M1c (IVB)	-	13/11/2017	0	60%; 5 + 5; PBx^**^	LVI, PNI, 10% necrosis	HT + CT	DOD (6)
	18							HT + CT	07/03/2018	3	20%; 5 + 5; Liver metastasis biopsy	5% necrosis	HT + CT	
14	19	80	6	8.80	18/07/2018	5 + 5	cT3bN0M0 (IIIB)	-	18/072018	0	5%; 5 + 5; PBx^**^	PNI	HT + RT, HT + CT	AWD (28)
15	20	74	10	28.00	06/08/2015	4 + 5	M1b (IVB)	HT + CT	12/12/2018	40	10%; 5 + 5; liver metastasis biopsy	10% necrosis	HT + CT	DOD (7)
16	21	72	10	83.00	31/05/2016	5 + 4	cT4N1M1b (IVB)	HT + CT	13/03/2019	33	20%; 5 + 5; TURP	LVI, PNI, 20% necrosis, MiNEN	CT	AWD (1)
17	22	74	10	34.00	31/01/2020	4 + 4	cT3bN1M1b (IVB)	-	19/02/2020	1	10%; 5 + 5; TURP	Cribriform, 10% necrosis, MiNEN	HT	AWD (19)
	23							HT	13/01/2021	11	40%; 5 + 5; TURP	10% necrosis, MiNEN	HT	
18	24	60	8	4.30	31/07/2020	4 + 5	cT3aN1M1c (IVB)	-	31/07/2020	0	60%; 4 + 5; PBx^**^	PNI, cribriform	HT + CT	AWD (24)
	25							HT + CT	19/02/2021	6	100%; 5 + 5; TURP	LVI	HT + CT	
19	26	67	4	5.66	30/09/2020	4 + 3	pT3bN0M0 (IIIB)	-	06/01/2021	4	10%; 5 + 4; RPLND	PNI, cribriform	RPLND	AWD (18)
20	27	61	4	12.14	06/04/2021	3 + 4	cT3bN1M0 (IVA)	HT + RT	07/12/2021	8	20%; 4 + 5; TURP	Cribriform	HT + RT	AWD (4)

%, percentage; *AWD*, alive with disease; *CT*, chemotherapy; *DOD*, died of disease; *HT*, hormone therapy; *mo*, months; *PCa*, prostate cancer; *ICI*, immune checkpoint inhibitor; *IDCP*, intraductal carcinoma of the prostate; *LVI*, lymphovascular invasion; *MiNEN*, mixed neuroendocrine and acinar prostate cancer; *N/A*, not applicable/not available; *PBx*, prostate biopsy; *PCa*, prostate cancer; *PGCC*, pleomorphic giant cell carcinoma; *PNI*, perineural invasion; *Ra-233*, radium-233; *RPLND*, radical prostatectomy with lymph node dissection; *RT*, radiation therapy; *sRT*, salvage RT; *TURP*, transurethral resection of prostate; *TURBT*, transurethral resection of bladder tumor

^*^All prostatic adenocarcinomas diagnosed on biopsy samples

^†^AJCC/TNM (8th edition) based on clinical or pathologic criteria

^‡^Refers to date of PGCC diagnosis as presented in this table

^§^Since the date of PGCC diagnosis

^**^PCa was diagnosed in the diagnostic prostate biopsy

Transurethral resection of the prostate (41%, 11/27) was the most common diagnostic sample, followed by prostate biopsies (33%, 9/27), radical prostatectomy specimens (15%, 4/27), liver metastases biopsies (7%, 2/27), and transurethral resections of bladder tumors (3.7%, 1/27).

**Fig. 1:**
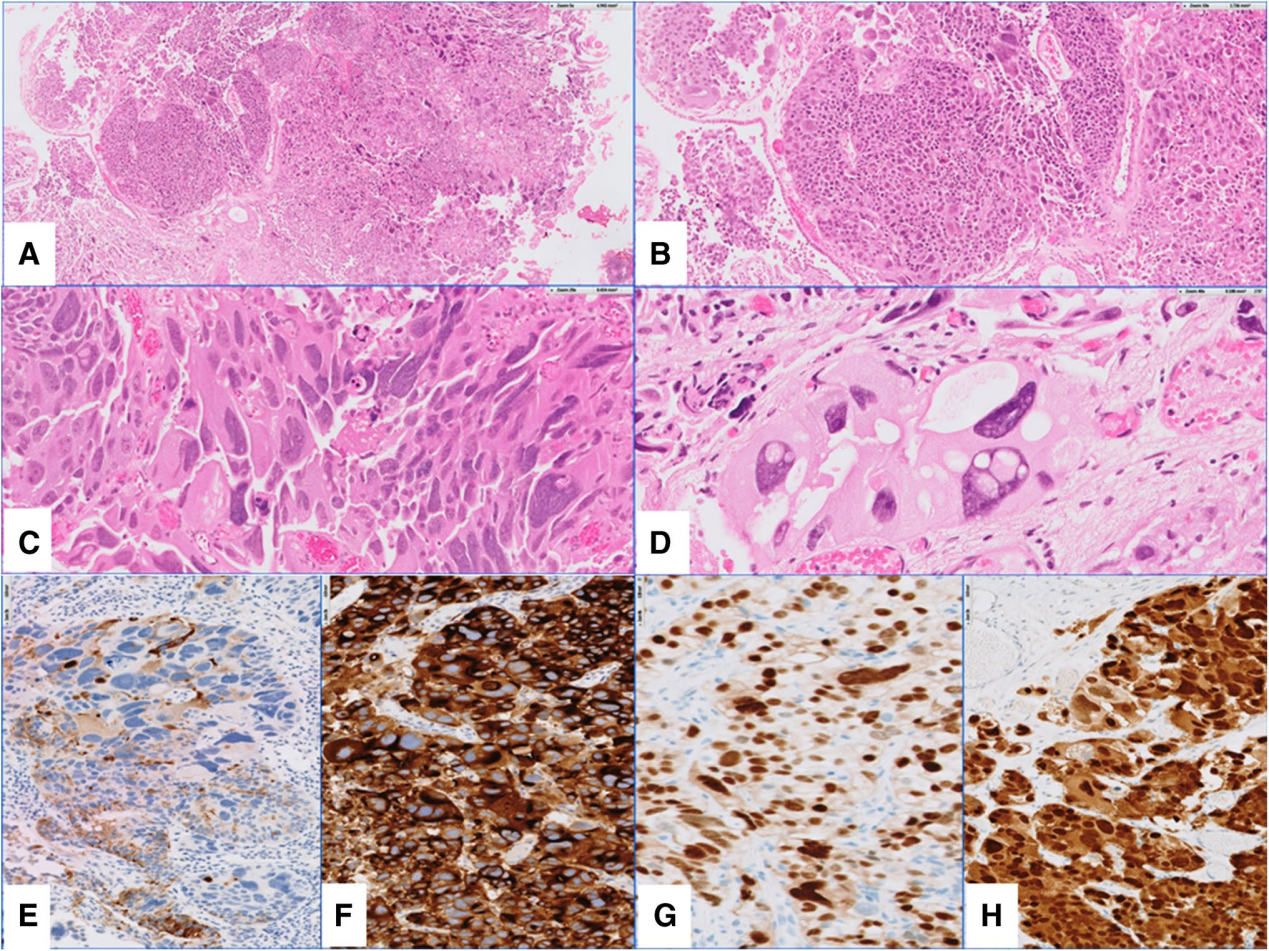
Transurethral resection of the prostate showing representative features of pleomorphic giant cell carcinoma with highly pleomorphic cells and largely variable hyperchromatic nuclei at low (**A**, **B**), medium (**C**), and high power (**D**) (**A**, **B**, **C**, **D** hematoxylin and eosin staining). Prostate lineage immunohistochemical markers are readily expressed by cells in pleomorphic giant cell carcinoma PSA (**E**), PSMA (**F**), NKX3.1 (**G**), and androgen receptor (**H**)

Giant bizarre cells with pleomorphic nuclei identified on H&E-stained glass slides typically characterized the histological appearance of PGCC (Fig. 1). Conventional prostatic acinar adenocarcinoma was initially found in all cases with ISUP grades 4 and 5. PGCC features were identified de novo in 11 (55%) patients and after previous treatment during tumor progression in 9 patients, with 16 ± 30 months (median 4 months) from the primary PCa diagnosis to the appearance of PGCC diagnostic features. Four patients had undergone previous treatment with hormone therapy (HT), 4 with HT and chemotherapy (CT), and 1 with HT and radiation therapy (RT). The pleomorphic component ranged 5–100% (24 ± 24%, median 10%) (Table 1).

**Fig. 2:**
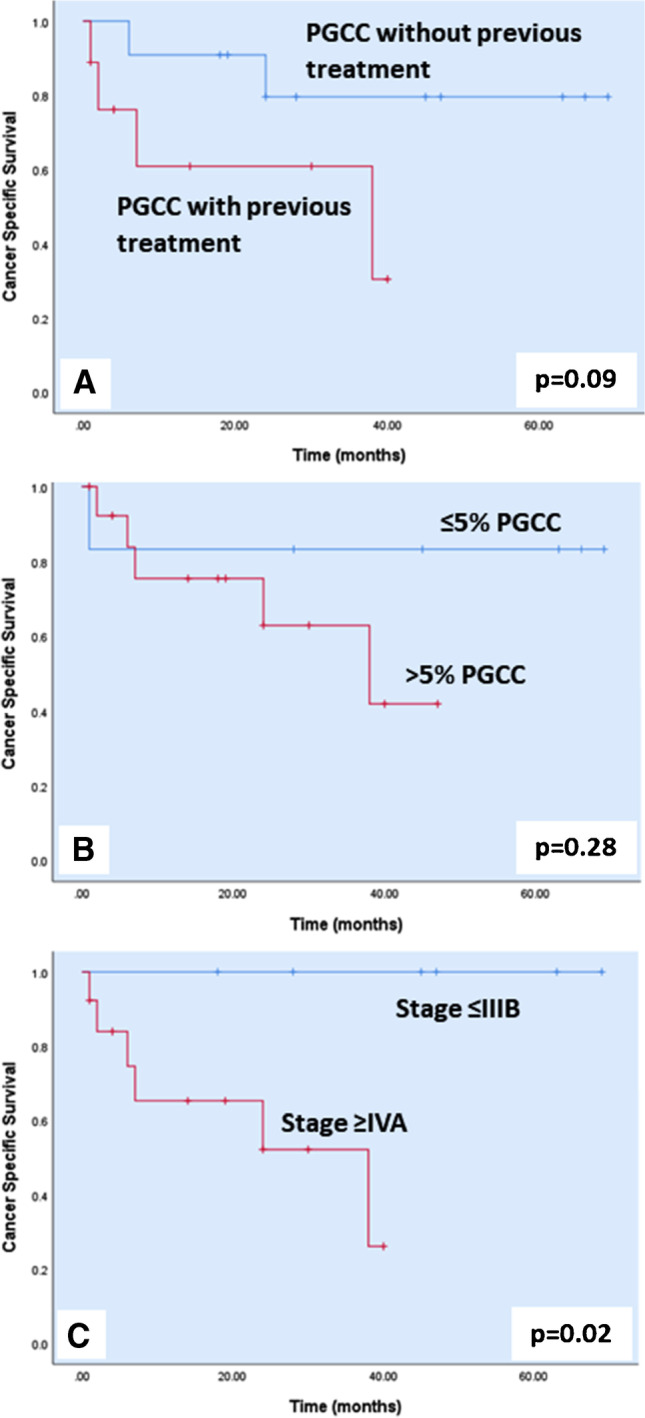
Kaplan–Meier plots showing cancer-specific survival differences for medical treatment (*p* = 0.09, **A**), the percentage of pleomorphic giant cell component (*p* = 0.28, **B**), and AJCC stage (*p* = 0.02, **C**) in the current series of pleomorphic giant cell carcinoma of the prostate

Regarding survival status, 14 (70%) patients were alive with the disease during follow-up (5 of those with de novo PGCC diagnosis), and 6 patients died of the disease. Our study also provided a comparison of survival between patients with PGCC diagnosis who had and had not been submitted to previous treatment(s) as well as between patients with ≤ 5% and > 5% of PGCC. Patients diagnosed earlier with lower TNM stage had longer survival than those diagnosed at a later T-stage or with metastatic disease (*p* = 0.02). The univariate survival analysis showed a trend towards longer survival in patients diagnosed with PGCC at the initial PCa diagnosis (58 ± 7 vs 25 ± 6 months, *p* = 0.09). The percentage of PGCC added no significance (51 ± 7 vs 27 ± 6 months, *p* = 0.21) (Fig. 2).

Immunohistochemistry was performed in 13 cases (Table 2) in the context of differential diagnosis. The expression of prostatic markers (PSA 5–60%, PSMA 20–80%, AR 90–100%, and NKX3.1 70–100%) identified PGCC as of prostatic origin and allowed to differentiate it from pleomorphic giant cell carcinomas from other organs. Ki-67 labeling ranged from 20 to 90%. P53 presented an overexpressed mutated pattern. None of the PGCC cases tested displayed PD-L1 positivity with 22C3 antibody. ERG was positive in case 10 (30%).

**Table 2:** *Immunohistochemistry of pleomorphic giant cell carcinoma and associated conventional carcinoma of the prostate

	Patient 3		Patient 5		Patient 6		Patient 7		Patient 13				Patient 15		Patient 16		Patient 17				Patient 18				
	Case 4		Case 6		Case 8		Case 10		Case 17		Case 18		Case 20		Case 21		Case 22		Case 23		Case 24		Case 25		
	C	P	C	P	C	P	C	P	C	P	C	P	C	P	C	P	C	P	C	P	C	P		C	P
PSA	40%	20%	70%	20%	20%	5%	30%	10%	70%	60%	-	-	-	-	0%	5%	40%	40%	100%	5%	20%	50%		20%	5%
PSMA	60%	40%	80%	20%	-	-	90%	80%	-	-	-	-	-	-	1%	0%	-	-	80%	60%	-	-		40%	20%
AR	-	-	-	-	-	-	95%	90%	100%	100%	100%	100%	-	-	0%	0%	-	-	100%	100%	100%	100%		100%	100%
NKX3.1	-	-	-	-	-	-	0%	0%	-	-	-	-	-	-	1%	1%	90%	70%	100%	100%	100%	100%		100%	100%
ERG	-	-	-	-	-	-	50%	30%	0%	0%	-	-	-	-	0%	0%	-	-	-	-	-	-		-	-
Ki-67	-	-	-	-	-	-	90%	80%	20%	20%	-	-	-	-	80%	90%	-	-	-	-	-	-		-	-
P53	-	-	90%	90%	-	-	10%	10%	70%	70%	-	-	-	-	-	-	-	-	-	-	-	-		-	-
Synaptophysin	-	-	-	-	1%	0%	20%	0%	5%	0%	0%	0%	-	-	0%	0%	-	-	10%	0%	-	-		0%	0%
PDL1	-	-	-	-	-	-	0%	0%	0%	0%	1%	0%	1%	0%	-	-	-	-	-	-	-	-		-	-

*C*, conventional prostate carcinoma component; *P* pleomorphic giant cell carcinoma component; *PGCC*, pleomorphic giant cell carcinoma

^*^In the context of differential diagnosis, selected cases were also stained for CK AE1/AE3 (case 4, 30% C, 70% PGCC), racemase (case 4, 70% C; 30% PGCC), CK20 (case 6, 20% C, 10% PGCC; case 10, negative in the C and in the PGCC), CK7 (case 6, 5% C, negative in the PGCC; cases 10 and 22, negative in the C and in the PGCC), CK5/6 (case 6, 20% C, negative in the PGCC; case 25, negative in the C and in the PGCC), MLH1/MSH2/MSH6/PMS2 (cases 7 and 20, preserved nuclear expression), CD138 (case 10, 40% C, 60% PGCC), INI1 (case 10, 25% C, 100% PGCC), e-cadherin (case 17, 100% in C and in PGCC), p120 (case 21, 70% C, 50% PGCC). Case 18 presented positive expression of AR and TOP2A and high expression of p4EBP1 and TUBB3. Beta-HCG (cases 4 and 6), inhibin (case 6), p40 (cases 8 and 10), p63 (cases 4 and 10), and GATA3 (cases 4, 10, and 22) were all negative in both conventional and PGCC components

Table 3 shows available molecular alterations in 3 samples based on Foundation One CDx®, (Roche Diagnostics, Penzberg, Germany) performed on patients 13 (case 17) and 15 (case 20) or OncoDEEP® (OncoDNA, Brussels, Belgium) performed in an additional metastatic biopsy sample of patient 13 (case 18). The molecular analysis showed a microsatellite-stable disease with low tumor mutation burden, and variable PTEN, PTCH1, KDM6A, ARv7, RB1, and PIK3CA loss/alteration, TP53 mutation, TMPRSS2-ERG fusion, and MYC, PIK3CB, RICTOR, or IRS2 amplification. Genomic analysis was undertaken in patients previously submitted to treatments (HT + CT in all of them), and all of them had a low mutational burden (4 or 5 mutations/Mb, respectively). Table 3 also includes relevant molecular data obtained from a recent literature search based on 16 reported cases.

**Table 3:** Reported gene molecular alterations in pleomorphic giant cell carcinoma of the prostate

Gene panel [ref.]	Case #	Tumor mutational burden	Microsatellite instability	Genomic alterations
UW-Oncoplex® [7]	Case 1	7 mut/Mb	MS-stable	BRCA2 mutation (p.L557 + LOH) TP53 alteration (p.M133_K139del + LOH) PTEN alteration (exon 6–8del + LOH)
	Case 2	1 mut/Mb	MS-stable	TP53 alteration (p.R273H)
	Case 3	4 mut/Mb	MS-stable	TP53 alteration (homozygous copy loss)
	Case 4	1 mut/Mb	MS-stable	None
	Case 5	62 mut/Mb	MSH2 (p.Q397* + c.1276 + 2 T > A)	BRAF mutation p.K601E PIK3CA mutation p.Y1021C TP53 alteration (p.G245S) PTEN alteration (p.N323Mfs*21)
	Case 6	0 mut/Mb	MS-stable	PIK3R1 mutation (p.P298_L303del) TP53 alteration (p.R175H + LOH)
	Case 7	5 mut/Mb	MS-stable	BRCA2 mutation (p.N1666Ifs*4 + LOH), NBN (p.P198Kfs*30 + LOH) TP53 alteration (p.A161T + LOH)
	Case 8	9 mut/Mb	MLH1 (homozygous copy loss)	BRCA2 mutation (exon 9 + part of exon 10 del) TP53 alteration (p.R248Q) PTEN alteration (K164Rfs*3 + LOH)
FISH and Archer® FusionPlex® Solid Tumor Kit [11]	Case 1	N/A	N/A	SND1-BRAF fusion
	Case 2	N/A	N/A	TMPRSS2-ERG fusion
	Case 3	N/A	N/A	ERG5’ deletion
	Case 4	N/A	N/A	TMPRSS2-ERG fusion
Ion Proton System with Torrent Sever® [5]	Case 1	N/A	N/A	PIK3CA mutation (c.3140A > G, pH1047R exon 20)
Current study Foundation One® or OncoDEEP®	Case 1^+^	4 mut/Mb	MS-stable	PTEN alteration (Y180fs*7) HGF amplification – equivocal MYC amplification PIK3CB amplification RICTOR amplification IRS2 amplification TP53 mutation (A159V)
	Case 2^&^	N/A	N/A	PTEN alteration (p.L180Efs*6) AR variant 7
	Case 3^+^	5 mut/Mb	MS-stable	PIK3CA (R93Q subclonal) PTCH1 (C699) PTEN loss TMPRSS2-ERG fusion KDM6A loss (exons 1–2) RB1 loss

^+^Foundation One® (cases 17 and 20 in Table 1 of the current manuscript)

^&^OncoDEEP® (case 18 in Table 1 of the current manuscript)

*FISH* fluorescence in situ hybridization; *LOH*, loss of heterozygosity; *Mb*, mega-base; *MS*, microsatellite; *mut*, mutations; *N/A*, not applicable/not available; *ref.*, reference number

Table 4 summarizes the results of the PubMed database search (www.pubmed.gov) conducted to identify all published cases of PGCC of the prostate in the English literature that was queried using the terms “prostate pleomorphic giant cell,” “giant cell carcinoma of the prostate,” “pleomorphic giant cell molecular alterations,” and “pleomorphic giant cell genomic analysis.” All relevant publications on the database describing cases of PGCC were retrieved. The literature search is current as of September 30th, 2022.

**Table 4:** Salient clinicopathologic features of pleomorphic giant cell carcinoma of the prostate obtained through literature search

Reference #	# of cases	Age (years)	Treatment prior to PGCC diagnosis	Sample type	PGCC %	Gleason score	Clinical outcome
[13]	1	70	None	Cystoprostatectomy	Extensive (% not provided)	9 (5 + 4)	DF (4 months after surgery)
[6]	2	1) 45 2) 77	None RT (?) for unknown reason and unspecified target	TURP Prostate at autopsy	Extensive (% not provided)	4 + 5, TURP 4 + 5, prostate at autopsy	DOD (9 months after the diagnosis) Dead on arrival (specimen obtained at autopsy)
[9]	6	59–76	HT RT N/A in 4 cases	PBx (3) RP TURP Urethral biopsy	5% in 4 cases 20% 70%	9 (4 + 5 or 5 + 4)	AWD (3 months, 12 months, and not specified), 3 cases AWD (metastases 2 years after diagnosis) AWD (large perineal recurrence after brachytherapy at 3 years) DOD (1 year of disease)
[17]	1	72	None	TURP	5%	5 + 4	AWD (not specified)
[15]	1	81	None	TURP	60%	9 (5 + 4)	DOD (12 months)
[2]	30	39–90	HT in 2 cases RT in 3 cases CT in 1 case None in 19 cases N/A in 4 cases	PBx (13) RP (1) TURP (7) Urethral/bladder biopsy (8) Orchiectomy (1)	1–5% in 21 cases 20% in 1 case N/A in 8 cases	9 or 10 7 (4 + 3) in 1 case	DOD (3–60 months after diagnosis) 10 cases
[7]	2	1) 66 2) 90	Possible HT in 1 case N/A in 1 case	TURP	< 5%	10	N/A
[11]	7	55–90	HT in 3 cases RT in 3 cases N/A in 1 case	TURBT/TURP Prostate Retroperitoneal metastasis biopsy	N/A	N/A	N/A
[5]	2	1) 52 2) 69	HT + CT HT + RT	TURBT and TURP TURBT	80% 98%	10	AWD (1 month after diagnosis) AWD (36 months after diagnosis)
Current study	27	51–84	HT + CT in 9 cases HT + RT in 1 case HT in 5 cases None in 12 cases	PBx (9) RP (4) TURP (11) TURBT (1) Liver biopsy (2)	5–100%	9–10	DOD (1–38 months after diagnosis) 8 cases AWD (1–69 months after diagnosis) 19 cases

%, percentage; *AWD*, alive with disease; *CT*, chemotherapy; *DF*, disease-free; *DOD*, died of disease; *HT*, hormone therapy; *N/A*, not applicable/not available; *PCa*, prostate cancer

*PBx*, prostate biopsy; *PGCC*, pleomorphic giant cell carcinoma; *RP*, radical prostatectomy; *RT*, radiation therapy; *TURBT*, transurethral resection of bladder tumor

*TURP*, transurethral resection of the prostate

## Discussion

Pleomorphic giant cell carcinoma (PGCC) of the prostate is a rare entity classified by the World Health Organization (WHO) 2022 as a subtype of prostatic acinar adenocarcinoma [1]. PGCC is defined by extreme nuclear atypia and pleomorphism, with characteristic bizarre multinucleated and mononuclear giant cells, usually with abundant cytoplasm and often atypical mitoses [1, 6, 14]. Cases in which PGCC is noted usually include a prostatic acinar adenocarcinoma component. PGCC is considered an aggressive neoplasm that has been described in several organs including the lung, pancreas, thyroid, hepatobiliary system, endometrium, kidney, and urinary bladder [9, 21, 23]. Thus, it explains the fact that PGCC is most frequently reported in the context of differential diagnosis considerations to establish the primary origin of PGCC. As suggested by Alharbi et al. [2], confirmation of the prostatic origin of PGCC may be accomplished by using prostatic marker immunohistochemistry, typically PSA, NKX3.1, PSMA, and androgen receptor. Our study confirms such results given the fact that our cases were positive for these markers [2]. In fact, PSA yielded lower positive results with a range of 5% to 20%; interestingly, the low expression should not be considered a negative expression of PSA since this is a common finding after different lines of therapy, as is the case of pleomorphic giant cell carcinoma of the prostate. Importantly, NKX3.1 and androgen receptor (0% to 100%) or PSMA (0% to 80%) immunohistochemical expression provides support for prostate origin in PGCC. Of relevance is the fact that immunohistochemistry in PGCC requires a panel approach using at least 2 prostate lineage markers, including PSA and NKX3.1 as initial selection and expanding the panel in case of need using androgen receptor and PSMA.

There seems to exist a frequent association between PGCC phenotype and prior chemo-, hormone-, and/or radiation therapy [3, 4, 8, 10, 12, 24]. Our study confirms the association with hormone therapy (present in all patients who had previously been submitted to treatment) with or without associated chemotherapy (in 44.4% of those, 4/9). However, our results also show that 55% of the PGCC diagnosed cases (11/20) were unrelated to previous therapy including those with a significant percentage of PGCC (5–60%) in the tumor. In fact, the current WHO classification states that PGCC is related to androgen deprivation or radiation therapy in many cases [1]. This statement is probably based on limited data available in the literature concerning the characteristics of PGCC of the prostate [1–24]. Our observation is, therefore, original and of relevance since it indicates that the aggressiveness of PGCC may be seen early before the hormone-independent status appears during the natural history of prostate cancer thus suggesting the for more aggressive therapy early on.

Our paper adds further information to previous pathology-related publications (Table 4) by providing the largest series of cases featuring extensive PGCC and by presenting a comparative survival analysis between different cohorts of patients. Interestingly, patients whose diagnosis of PGCC was made after PCa treatment(s) had shorter cancer-specific survival than those who were diagnosed de novo (early onset of PGCC). This leads us to hypothesize that previous treatment(s) might not only act as a trigger but also as a promoter of PGCC, thus adding to the aggressiveness of the disease. Nonetheless, the survival analysis in the current study may be hampered due to the limited follow-up (mean 24, median 18 moths) associated with our patients. Another important observation is related to the amount of PGCC component in prostate cancer. In our series, the percentage of PGCC was not a significant predictor of cancer-specific survival, thus indicating that even a minor component of PGCC portends high aggressiveness to prostate cancer. All patients included in the study presented with an advanced stage PCa (63% IVB, 5% IVA, 26% IIIB, and 5% IIIA according to AJCC/TNM, 8th edition) [25], which reflects the biological aggressiveness of the disease from the very early onset of the disease and is significantly associated with poor survival.

The knowledge on molecular alterations in PGCC is limited [5, 7, 11]. A PubMed database literature search identified molecular data on 16 previously reported cases (Table 3). In line with them, our comprehensive molecular analysis based on genomic analysis of three cases identified similar findings, mostly describing a microsatellite-stable disease with low tumor mutation burden, and variable PTEN, PTCH1, KDM6A, ARv7, and PIK3CA loss/alteration, TP53 mutation, TMPRSS2-ERG fusion, and MYC, PIK3CB, RICTOR, or IRS2 amplification. BRCA2 mutations were not present in our series but occasional examples may be seen in the literature (3 cases, 19% of reported cases with molecular analysis), a finding with therapeutic implications (Table 3). In the future, these findings may be of relevance to identifying novel therapeutic targets useful in a dismal disease as is the case of PGCC. In particular, our study suggests that patients diagnosed with PGCC may benefit from PI3K/AKT/mTOR signaling pathway small-molecule inhibitors [19, 20, 22].

Our study also shows that PGCC might integrate the so-called aggressive variants of prostate cancer [8] due to the fact that it included patients with at least one of the seven criteria detailed by Aparicio et al. [3] and Vlachostergios et al. [10], namely, bulky high-grade Gleason score (Gleason score of at least 8), tumor mass in prostate/pelvis, and the presence of exclusively visceral metastasis. Aggressive variants of prostate cancer are clinically characterized by a rapidly progressive disease course [8]. Our study suggests that PGCC appears in an early stage not related to the appearance of neuroendocrine phenotype, a finding that deserves additional research on the status of androgen receptor, most probably placing PGCC as an androgen-indifferent status prostate cancer or as a transitive status from androgen indifferent to androgen-independent status prostate cancer [4, 8]. A status that might be related to the important methylation alterations is seen in aggressive variants of prostate cancer [19].

Concomitantly, lineage plasticity has emerged as a relevant explanatory theory of treatment resistance in prostate cancer. It occurs in up to 20% of advanced PCa patients resulting in important clinical and therapeutic implications [3, 4, 10, 12, 19]. Lineage plasticity refers to a process in which cells are capable of reprogramming their identity by acquiring alternative characteristics, which may or may not be reversible [4, 19]. According to this hypothesis, treatment resistance might derive from an intermediate stem-like state, an epithelial–mesenchymal transition appearance, or from direct trans-differentiation in which cells acquire new characteristics whose reversibility is still equivocal. Therefore, we hypothesized that PGCC could be a morphological way to identify the transitive status between androgen-indifferent and androgen-independent prostate cancer; this hypothesis needs to be further investigated. Studies to elucidate the potential role of trans-differentiation in PGCC phenotype appearance are on their way.

## Conclusions

Pleomorphic giant cell carcinoma of the prostate represents a complex, rare, and aggressive entity, which may be diagnosed ab initio in the context of prostate cancer with a high Gleason score (ISUP 5) or during follow-up of prostate cancer treated with a combination of radiation-, hormone-, and/or chemotherapy. It has poor cancer-specific survival, especially in patients in which PGCC appears in the post-treatment setting, a fact probably due to the achievement of a hormone-independent status in which a novel therapeutic approach might be necessary. Early recognition of this entity could help in providing more efficacious treatment, and therefore, pleomorphic giant cell carcinoma is a subtype of PCa worth reporting.

## Data Availability

All data related to this manuscript is available upon request.
